# Face to face interactions in chimpanzee (*Pan troglodytes*) and human (*Homo sapiens*) mother–infant dyads

**DOI:** 10.1098/rstb.2021.0478

**Published:** 2023-04-24

**Authors:** Federica Amici, Manuela Ersson-Lembeck, Manfred Holodynski, Katja Liebal

**Affiliations:** ^1^ Faculty of Life Sciences, Institute of Biology, Human Biology and Primate Cognition, Leipzig University, Talstrasse 33, 04103 Leipzig, Germany; ^2^ Department of Comparative Cultural Psychology, Max Planck Institute for Evolutionary Anthropology, 04103 Leipzig, Germany; ^3^ Department of Education and Psychology, Comparative Developmental Psychology, Freie Universität Berlin, Habelschwerdter Allee 45, 14195 Berlin, Germany; ^4^ Faculty of Psychology, Institute of Psychology in Education, University of Münster, Fliednerstrasse 21, 48149 Münster, Germany

**Keywords:** face-to-face interactions, chimpanzees, humans, mother–infant dyads

## Abstract

Human mothers interact with their infants in different ways. In Western, educated, industrialized, rich and democratic (WEIRD) societies, face-to-face interactions and mutual gazes are especially frequent, yet little is known about their developmental trajectories and if they differ from those of other primates. Using a cross-species developmental approach, we compared mother–infant interactions in 10 dyads of urban humans from a WEIRD society (*Homo sapiens*) and 10 dyads of captive zoo-based chimpanzees (*Pan troglodytes*), when infants were one, six and 12 months old. Results showed that face-to-face interactions with mutual gaze events were common in both groups throughout the infant's first year of life. The developmental trajectories of maternal and infants’ looks partially differed between species, but mutual gaze events were overall longer in humans than in chimpanzees. Mutual gazes were also more frequent in humans, peaking at six months in humans, while increasing with age in chimpanzees. The duration and frequency of mutual gazes varied across contexts in both groups, with mutual gazes being longer during caring/grooming and feeding contexts. These findings confirm that some aspects of early socio-cognitive development are shared by humans and other primates, and highlight the importance of combining developmental and cross-species approaches to better understand the evolutionary roots of parenting behaviour.

This article is part of a discussion meeting issue ‘Face2face: advancing the science of social interaction’.

## 1. Introduction

Human communication and cognition seem to be unique and different from those of other animals in many ways. To investigate the differences and similarities across species, researchers usually compare humans with other apes to identify those behaviours unique to humans or those shared with other species. Only more recently, researchers have also started to compare the developmental trajectories of cognitive and communicative skills across species, as pace and patterns of their emergence might vary between humans and other primates [1–4]. A combination of cross-species and developmental approaches thus enables the assessment of inter-species differences in the temporal emergence and pattern of communicative and cognitive skills during ontogeny, shedding light on the functions of developmental change and the evolutionary origins of human communication and cognition [1]. However, despite the importance of this approach, few studies manage to integrate the developmental and comparative perspectives, mostly because of time and budget constraints, and of the intrinsic difficulties of longitudinally following a large enough number of individuals across species. The present study aimed to combine the comparative and longitudinal approach to investigate developmental trajectories of face-to-face interactions and mutual gaze in mother–infant pairs of a selected group of humans and chimpanzees during the first year of an infants’ development.

### (a) Parenting behaviour

In mammals, uniparental female care is the most widespread condition, as infants are mostly fed by lactation during the first phases of their development and therefore heavily depend on their mothers [5]. Indeed, several studies have shown that by nurturing, protecting and caring for their infants, mothers increase their chances of survival [6]. These forms of parenting are especially important in species that have long lifespans, slow pace of development and large brains, like humans and great apes, as they have extended periods of juvenility, and maternal investment is crucial to increase infants’ fitness (e.g. [7–9]). In turn, these conditions provide extended opportunities for social learning, and also for the acquisition of socio-cognitive and emotional skills that are fundamental for complex cognitive processes (e.g. theory of mind; see [10] and [1], for reviews).

### (b) Face-to-face interactions in human communities

In humans, there is a huge variation in the way in which mothers provide care to their infants. The need to nurture and protect infants, for instance, is a universal belief that all communities share [11,12], but different societies can differ in other beliefs, or in the way they express them [13]. In several societies, for instance, mothers show proximal parenting styles and frequently interact with their infants through body contact and tactile stimulation, whereas in other societies face-to-face interactions are among the most important forms of social interactions between mothers and infants (e.g. [14–18]). In Western, educated, industrialized, rich and democratic (WEIRD) societies [19], in particular, face-to-face interactions are thought to provide infants with crucial competencies, foster affect regulation and coordination, and promote the development of sense-of-self and linguistic skills (e.g. [20,–28]). Therefore, face-to-face interactions and mutual gazes are especially frequent in WEIRD societies: mothers and infants often face each other, and engage in a variety of non-verbal means of communication, including facial expressions and eye contact (e.g. [18,29]). Through mutual gaze, mothers from WEIRD societies are thought to establish a communicative context with their infants, promoting the establishment of joint attention [30]. By contrast, face-to-face interactions and mutual gaze are often much scanter in other societies [14,15,31], where joint engagement may be expressed in other modalities, without the need to establish reciprocal visual contact [18].

### (c) Face-to-face interactions in chimpanzees

In species other than humans, mothers may also engage in face-to-face interactions and mutual gazes with their infants (e.g. [32]). During the first year of an infants’ life, for instance, chimpanzees do not only show very basic components of parenting behaviour like humans such as primary care, body stimulation or contact, but they also engage in face-to-face interactions and mutual gazes, despite important variation across study groups and individuals [18,24,33]. Moreover, as for humans, chimpanzees from an early age prefer maternal faces over less familiar ones [34] and faces with direct gaze over averted-gaze faces [35]. Therefore, despite differences in important aspects of socio-cognitive development at older ages, some basic features of face-to-face interactions and mutual gaze might be shared by humans and other species (see [1,33]). Clearly, there is also abundant variation across chimpanzee individuals and groups in the frequency of mutual gazes and face-to-face interactions. As for humans, for instance, chimpanzee mothers more often engaging in physical contact and tactile interactions with their offspring are also less likely to engage in mutual gaze with them [24].

To date, what we know about mutual gaze in chimpanzee mother–infant interactions is still limited. In humans, for instance, mutual gaze is thought to have an ostensive function, increasing children's receptivity and facilitating transfer of knowledge [36,37], and to have an important role in fostering social attunement [38,39], at least in WEIRD societies. By contrast, it is yet unclear whether and to what extent mutual gaze might serve a similar function in other primate species. Observations of captive chimpanzees have shown that mothers engage in mutual gaze with their infants from the second week of age [33]. Depending on the study, the previous raising histories of chimpanzees and the age of the infants (up to three months), the frequency of mutual gazes varied between eight to almost 30 occurrences per hour of visible time [24,33]. Importantly, mutual gaze repeatedly occurred in positive contexts, including social play or grooming [24,33], suggesting that face-to-face interactions and mutual gaze can be embedded in positive social context also in species other than humans. Indirect comparisons with studies on humans, however, also suggest important differences in other aspects of mutual gazes, with their duration being probably longer in humans than in chimpanzees, and maternal looks being overall more frequent in humans than in other species (for a discussion, see [24]).

Moreover, little is still known on the qualitative aspects of face-to-face interactions in chimpanzees, including the context in which these interactions occur. In both humans and chimpanzees, for instance, mutual gaze events appear to be especially frequent in positive, non-agonistic contexts (see [24]). However, it is not yet clear which specific contexts are linked to longer, more frequent instances of mutual gaze. Grooming or caring activities often imply that mothers direct their gaze towards their infants, which might facilitate the occurrence or increase the duration of mutual gazes. In chimpanzees, however, Bard *et al*. [24] found that the frequency of mutual gazes was not related to the time mothers spent inspecting, restraining, soothing or playing with their infants, and that the frequency of mutual gaze events was lower when mothers spent more time grooming or cradling their infants. Furthermore, the few existing studies on face-to-face interactions in chimpanzees focus on very young infants (i.e. up to three months), but do not address how gazing patterns might change with infant's increasing age (and mobility), although gazing patterns are known to drastically change over the first months of an infants’ life, as a result of maturational processes and cultural factors (e.g. eye contact decreases as children begin to engage in exploration of objects; [40]).

### (d) Aims of the study

The aim of this study was to investigate face-to-face interactions and mutual gaze during natural interactions between mothers and infants in two different study groups: captive zoo-based chimpanzees, who were socially housed with their conspecifics, and humans from a WEIRD society living in two German cities. Clearly, these two study groups are not necessarily representative of the whole species, and generalization to other individuals of the same species should not be assumed [41]. However, by comparing the developmental trajectories of mutual gaze in the two study groups, it is possible to acquire novel information about possible differences in the developmental trajectories of mutual gaze under specific conditions (e.g. captivity, WEIRD settings), and thus on the conditions that might explain this variation, and the potential of the species. Moreover, both study groups are quite representative for members of humans and chimpanzees that have been recruited for developmental research up to now. Unlike previous research, we aimed to directly compare the two study groups by using the same methodological approach. Furthermore, unlike other studies, we extended existing research beyond infants’ age of three months [24,33] and focused on both quantitative (i.e. frequency and duration of infants’, maternal looks and mutual gaze events) and qualitative aspects (i.e. context in which these behaviours occurred) of face-to-face interactions and mutual gaze when infants were one, six and 12 months old. Based on existing literature, we hypothesized that mutual gaze would be present in both study groups from early on, but that there would be quantitative and qualitative differences in its use between groups during an infants’ development. In particular, we predicted that the duration of maternal looks, infants’ looks and mutual gazes would be overall longer, and that the frequency of mutual gazes would be overall higher in WEIRD urban humans than in captive zoo-based chimpanzees (prediction 1). However, we also predicted that the duration of maternal looks, infants’ looks and mutual gazes, and the frequency of mutual gazes, would decrease in both study groups during the first year of development, as infants become more independent and move more (prediction 2). Finally, we predicted that the duration of maternal looks, infants’ looks and mutual gazes, and the frequency of mutual gazes, would vary across contexts in both study groups, with events being longer and more frequent when mothers engage in grooming or caring activities with their infants, as compared to other contexts (prediction 3).

## 2. Methods

### (a) Study subjects

For this study, we longitudinally followed 20 mother–infant pairs belonging to the superfamily Hominoidea, including 10 WEIRD humans from two cities in Germany (*Homo sapiens*) and 10 captive zoo-based chimpanzee pairs (*Pan troglodytes*). Half of the infants in each species were females, and half were males. For a complete list of the study subjects, please refer to table 1. Human pairs were recruited from two large cities in Germany (Berlin, Leipzig) using the participant pool of the Excellence cluster ‘Languages of Emotion’ or through study advertisements posted in public areas of the Freie Universität Berlin, among pregnant women in their third trimester or women with infants younger than one month. We only included women with no signs of post-natal depression and who had had a full-term delivery. Research was conducted in accordance with the ethical recommendations of the German Psychological Association (Deutsche Gesellschaft für Psychologie, DGPs): mothers were informed about the study content and procedure and provided written consent to participate in the study. The data (videos and coded behaviours) were pseudonymized and stored on a secure server at Freie Universität Berlin, and only project members had access to the data.

**Table 1.** For WEIRD urban humans (i.e. humans) and captive zoo-based chimpanzees (i.e. chimpanzees), identity and sex/gender of the study subjects, and individual observational effort at one, six and 12 months of infants’ age (including median with lower and upper quartiles for the two study groups).

study group	subject	sex	observational effort (in minutes): both faces are visible / coded video		
			one month	six months	12 months
humans	An	male	49 / 60	20 / 60	22 / 60
	Hd	female	35 / 61	40 / 60	35 / 60
	Hl	female	47 / 62	26 / 60	12 / 60
	Hn	female	0 / 61	1 / 34	5 / 65
	Jl	female	13 / 60	40 / 60	13 / 60
	Jr	male	34 / 64	31 / 60	16 / 60
	Ld	male	26 / 60	16 / 60	9 / 60
	Ls	male	29 / 64	28 / 61	6 / 60
	Mn	female	27 / 61	20 / 60	7 / 60
	Pp	male	13 / 60	25 / 60	8 / 60
			28 (16–35) / 61 (60–62)	26 (20–30) / 60 (60–60)	11 (7–15) / 60 (60–60)
chimpanzees	Azibo	male	11 / 15	14 / 28	13 / 31
	Bangolo	male	12 / 25	10 / 15	5 / 15
	Kara	female	33 / 183	38 / 181	4 / 183
	Kofi	male	32 / 183	75 / 185	1 / 183
	Lobo	male	35 / 62	24 / 46	13 / 42
	Lome	male	16 / 59	26 / 42	7 / 28
	Mora	female	20 / 186	60 / 182	0 / 0
	Nayla	female	50 / 181	72 / 181	0 / 0
	Tai	female	17 / 34	18 / 45	11 / 43
	Yara	female	30 / 189	34 / 181	20 / 180
			25 (16–33) / 122 (40–183)	30 (20–55) / 114 (43–181)	6 (2–13) / 37 (18–146)

Captive zoo-based chimpanzee pairs were observed at the Leipzig Zoo, at the Kristiansand Dyrepark and at the Osnabrück Zoo. Chimpanzees lived in their social groups in indoor and outdoor facilities with climbing materials and enrichment objects. The procedures were purely observational and did not require any change of the daily routine of the apes. Therefore, the study did not require a specific ethical approval, but it was approved by the participating zoos (Leipzig Zoo, Kristiansand Dyrepark, Osnabrück Zoos). These zoos fulfilled the terms of the WAZA Code of ethics and animal welfare [42], the Guidelines for the Treatment of Animals in Behavioural Research and Teaching of the Association for the Study of Animal Behaviour [43] and the EAZA Minimum Standards for the Accommodation and Care of Animals in Zoos and Aquaria [44].

### (b) Behavioural observations and coding

We conducted behavioural observations using focal animal sampling [45] and video-recorded them with a digital video camera (Panasonic, HDC-HS30). In both study groups, we conducted observations when the infant was supposed to be mostly active, during different daily activities, requesting no changes in the daily routine from participants. In the human study group, mother–infant pairs were video-recorded during home visits in three sessions (with the exception of one infant), when the infant was one, six and 12 months old (±8 days). These age points were selected because they represent major developmental milestones in human infants. When they are one month old, for instance, humans are still on their way to adapt to the extrauterine and have just started social engagement. At six months, instead, they have already established a strong relationship with their mother and perform exploratory behaviour, whereas at 12 months they have already mastered joint attention and start engaging in prosocial and cooperative activities. As developmental milestones are not as clearly identified in chimpanzees (for a first systematic study in wild chimpanzees, see [46]), we used the same age points for both species, although they may clearly reflect different developmental phases.

Human infants were observed at home only with their mother, but the father could also be present (although usually in another room). In the chimpanzee study group, mother–infant pairs were observed when infants were one, six and 12 months old, during the official opening hours of the zoo, from the visitors’ area, to avoid affecting animals’ behaviour. As the individuals lived in social groups, they were observed in the presence of other conspecifics beyond their parents. For practical reasons, the length of the chimpanzee videos varied from 5 to 60 min, so that multiple sessions were conducted for each subject and age. Because of the limited availability of some infant chimpanzees and the death of one chimpanzee infant (Mora), the observational effort across individuals and developmental phases of the chimpanzees is not identical (table 1).

Gaze behaviour of mother–infant interactions was coded from the videos for each of the 20 mother–infant pairs (10 WEIRD urban human dyads and 10 captive zoo-based chimpanzee dyads). When both faces were visible in the videos, we coded all instances and exact durations of: (i) maternal looks towards the infant, (ii) infant looks towards the mother, and (iii) mutual gazes (i.e. infants and mothers simultaneously looked at each other, with their faces aligned). Looks and mutual gazes were recorded whenever mothers and/or infants clearly moved their face and/or eyes in the direction of the partner, regardless of their duration. Given that chimpanzees can glance very shortly at each other (e.g. [47,48]), we did not set a minimum duration time as threshold.

The situational context was coded for each look or mutual gaze event. We chose among one of the following categories, which were exhaustive and mutually exclusive: (i) caring or grooming (i.e. mothers were cleaning or providing for the hygienic needs of their infants; hereafter caring), (ii) eating or feeding (i.e. infants were eating or receiving food from their mothers; hereafter feeding), (iii) locomotion (i.e. mothers and infants were climbing or walking on two or four limbs), (iv) resting or sleeping (i.e. infants and mothers were sitting or lying or in a state of sleep; hereafter resting), (v) social play (i.e. mothers and infants engaged in play activities with each other), (vi) solitary play (i.e. infants engaged in object exploration), or (vii) other activities (i.e. if none of the definitions above applied; for a similar classification of context, see [49]). Additionally, for each mutual gaze event we also coded whether the individuals were in body contact. For a detailed overview of the individual observational effort across development, please refer to table 1. ‘To assess inter-observer reliability, an observer naïve to the aims of the study recoded 1 h of video for each study group and developmental phase (i.e. 6/79 h, or 7.6% of the videos). Inter-observer reliability was very good for all the variables coded (i.e. Spearman's correlation for duration of maternal looks: *ρ* = 0.96, *n* = 290; for duration of infants’ looks: *ρ* = 0.95, *n* = 56; for duration of mutual gazes: *ρ* = 0.96, *n* = 62; for frequency of mutual gazes: *ρ* = 0.97, *n* = 13; Cohen's *k* for context: *k* = 0.91, *n* = 408; for body contact *k* = 0.97, *n* = 387; all *p* < 0.001).

### (c) Statistical analyses

We conducted all analyses in R (R Core Team, version 4.0.2 [50]), using generalized linear mixed models (GLMM, [51]) with the ‘glmmTMB’ package [52]. We conducted three models, to assess variation in the duration of looks by mothers and infants (model 1), in the duration of mutual gazes (model 2) and in the frequency of mutual gazes (model 3). Models were built in the following way. First, we built full models containing test predictors, random effects, controls and offset terms. In models 2 and 3, we included as test predictor the two-way interaction of study group with age, in order to assess if our dependent variable varied across developmental phases and study groups. In model 1, instead, we used the three-way interaction of study group, age and whether the look was produced by the mother or the infant, to also assess potential differences in the duration of looks by mothers and infants across developmental phases and study groups. Please note that all three- and two-way interactions also included the corresponding main effects, and three-way interactions also included all possible two-way interactions, which were not interpreted. In all models, we also included context as test predictor in order to assess variation across contexts, pair identity as random effect, and session identity as random effect nested in pair identity, in order to control for lack of independency [53]. In models 1 and 2, the dependent variable (i.e. duration) was right-skewed, so we first log-transformed it to obtain a normal distribution, and then used a Gaussian distribution, controlling for the distance between mother and infant (i.e. whether they were in body contact or not). In model 3, the dependent variable was the number of mutual gazes produced during the time in which both mother and infant faces were visible, which was operationalized by using a Poisson distribution, and including the log-transformed observational effort (i.e. the time in which both faces were visible) as the offset term.

Each of the three full models was then compared with likelihood ratio tests (LRTs) to a corresponding null model only containing random effects, controls and offset terms [54]. If the comparison was significant, we used the drop1 function to assess which test predictors had a significant effect. When interactions were not significant, we ran the model again, but only including their main effects (i.e. only removing the interaction term). No other predictor was removed from the models. In the case of significant categorical predictors with more than one level (i.e. age and context), we used the package emmeans to conduct *post hoc* tests with Tukey adjustments [55]. Finally, for each model, we checked residual diagnostics and overdispersion using the ‘DHARMa’ package [56] and multi-collinearity using the ‘performance’ package [57]. Multi-collinearity was low in all models (maximum variance inflation factors across models = 1.27; [58]).

## 3. Results

The duration and frequency of mutual gazes varied between study groups and across developmental phases (table 2). Mutual gazes were mostly exchanged when mothers and infants were in body contact, especially in captive zoo-based chimpanzees (table 2). In both study groups, and across all developmental phases, the percentage of mutual gaze events initiated by infants (i.e. infants started looking when mothers were already looking) was the highest (table 2).

**Table 2.** For WEIRD urban humans (i.e. humans) and captive zoo-based chimpanzees (i.e. chimpanzees) at one, six and 12 months of infants’ age, median (with lower and upper quartiles) of the duration and frequency of mutual gaze (MG) events; percentage of MG occurring when mothers and infants were in body contact; and percentage of MG initiated by mothers, infants or simultaneously by both.

	humans			chimpanzees		
	one month	six months	12 months	one month	six months	12 months
MG duration (s)	4.3 (1.4–10.00)	3.0 (1.2–6.5)	1.5 (0.8–2.6)	2.1 (1.2–4.1)	1.3 (0.6–2.8)	1.8 (1.1–7.0)
MG frequency (per minute)	1.1 (0.8–1.3)	2.7 (2.5–3.4)	1.9 (1.3–2.7)	0.1 (0.0–0.2)	0.1 (0.0–0.3)	0.4 (0.3–0.8)
MG in body contact	97%	75%	41%	100%	97%	95%
MG initiated by mothers–infants–both	37-40-23%	36-44-20%	16-62-23%	34-55-11%	37-44-20%	34-55-11%

**Figure 1.**
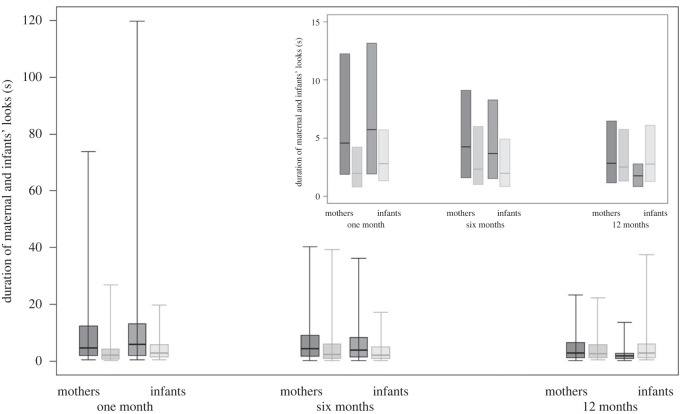
For WEIRD urban humans (in dark grey) and captive zoo-based chimpanzees (in light grey), central lines represent the median duration of looks (in seconds) performed by mothers and infants when infants were one, six and 12 months old. The horizontal ends of the box represent the 75% and 25% quartiles. The ends of the whiskers represent the 97.5% and 2.5% quartiles, and are not depicted in the smaller panel (which is otherwise identical to the larger one), to visually facilitate the detection of differences across study groups and developmental phases. In WEIRD urban humans, infants’ looks were longer at one and six months than at 12, and mothers’ looks were longer at one month than at 12; mothers’ looks at one month were longer in WEIRD urban humans than in captive zoo-based chimpanzees, and in WEIRD urban humans at 12 months, mothers’ looks were longer than infants’ looks (model 1).

In model 1, we tested whether the duration of looks by mothers and infants varied across developmental phases in the two study groups, and across contexts. The full model significantly differed from the null model (GLMM, *χ*^2^_17_ 151.50, *p* < 0.001), with both the three-way interaction (*p =* 0.006) and context (*p <* 0.001) having a significant effect (table 3). In particular, *post hoc* tests revealed differences along developmental phases, but only in WEIRD urban humans, with infants’ looks being significantly longer at one and six months than at 12 (one versus 12 months, and six versus 12 months, both *p <* 0.001), and maternal looks also being longer at one than at 12 months (*p =* 0.011; figure 1). *Post hoc* tests further revealed differences between study groups, but only for the duration of maternal looks at one month of age, which were significantly longer in WEIRD urban humans than in captive zoo-based chimpanzees (*p <* 0.001; figure 1). Finally, *post hoc* tests revealed differences in the behaviour of mothers and infants, but only in WEIRD urban humans at 12 months, when the duration of maternal looks was significantly longer than the duration of infants’ looks (*p <* 0.001; figure 1). Concerning context effects, *post hoc* tests showed that the duration of infants’ and maternal looks was generally longer during caring than during locomotion, resting, social and solitary play (all *p* < 0.001), and it was also generally longer during feeding, resting, social and solitary play than during locomotion, and during feeding than social play (all *p* < 0.010; see the electronic supplementary material).

**Table 3.** Results of the three models run, including estimates, standard errors (s.e.), confidence intervals (CIs), likelihood ratio tests (LRT), degrees of freedom (d.f.) and *p* values for each test and control predictor (in italics), with the reference category in parentheses.

Model	estimate	s.e.	2.5% to 97.5% CI	*LRT*	d.f.	*p*
model 1: duration of mothers' and infants' looks						
intercept	8.17	0.25	7.68 to 8.67	–	–	–
agent (mother) * study group (human) * age (six months)	−0.52	0.29	−1.09 to 0.06	10.14	2	0.006*
agent (mother) * study group (human) * age (12 months)	0.29	0.34	−0.37 to 0.95			
agent (mother) * study group (human)	0.39	0.25	−0.10 to 0.89	–	–	–
agent (mother) * age (six months)	0.59	0.26	0.07 to 1.11	–	–	–
agent (mother) * age (12 months)	0.22	0.30	−0.38 to 0.82			
study group (human) * age (six months)	−0.15	0.30	−0.76 to 0.45	–	–	–
study group (human) * age (12 months)	−1.03	0.34	−1.70 to −0.35			
agent (mother)	−0.34	0.23	−0.80 to 0.11	–	–	–
study group (human)	0.71	0.28	0.16 to 1.25	–	–	–
age (six months)	−0.21	0.26	−0.72 to 0.31	–	–	–
age (12 months)	−0.02	0.29	−0.60 to 0.56			
context (eating)	−0.25	0.10	−0.44 to −0.06	74.53	6	<0.001*
context (locomotion)	−0.88	0.11	−1.08 to −0.67			
context (other)	−0.49	0.20	−0.89 to −0.08			
context (resting)	−0.41	0.09	−0.57 to −0.24			
context (social play)	−0.52	0.11	−0.73 to −0.31			
context (solitary play)	−0.46	0.11	−0.66 to −0.25			
*body contact*	0.03	0.06	−0.09 to 0.15	0.25	1	0.620
model 2: duration of mutual gazes						
intercept	8.03	0.22	7.59 to 8.47	–	–	–
study group (human)	0.48	0.18	0.12 to 0.83	6.30	1	0.012*
age (six months)	−0.44	0.17	−0.76 to −0.11	13.43	2	0.001*
age (12 months)	−0.71	0.19	−1.08 to −0.34			
context (eating)	−0.10	0.18	−0.44 to 0.25	15.82	6	0.015*
context (locomotion)	−0.71	0.21	−1.11 to −0.31			
context (other)	−0.81	0.43	−1.66 to 0.03			
context (resting)	−0.30	0.15	−0.60 to 0.00			
context (social play)	−0.26	0.18	−0.61 to 0.08			
context (solitary play)	−0.37	0.19	−0.74 to 0.00			
*body contact*	−0.15	0.10	−0.34 to 0.05	2.11	1	0.147
model 3: frequency of mutual gazes						
intercept	−15.81	0.34	−16.47 to −15.14	–	–	–
study group (human) * age (six months)	−0.18	0.47	−1.10 to 0.74	8.77	2	<0.012*
study group (human) * age (12 months)	−1.37	0.51	−2.36 to −0.38			
study group (human)	2.75	0.41	1.95 to 3.55	–	–	–
age (six months)	1.09	0.38	0.35 to 1.83	–	–	–
age (12 months)	1.85	0.42	1.02 to 2.67			
context (eating)	0.32	0.10	0.11 to 0.52	436.43	5	<0.001*
context (locomotion)	−0.83	0.14	−1.12 to −0.55			
context (resting)	1.11	0.09	0.93 to 1.29			
context (social play)	0.27	0.11	0.06 to 0.48			
context (solitary play)	−0.22	0.12	−0.45 to 0.02

**Figure 2.**
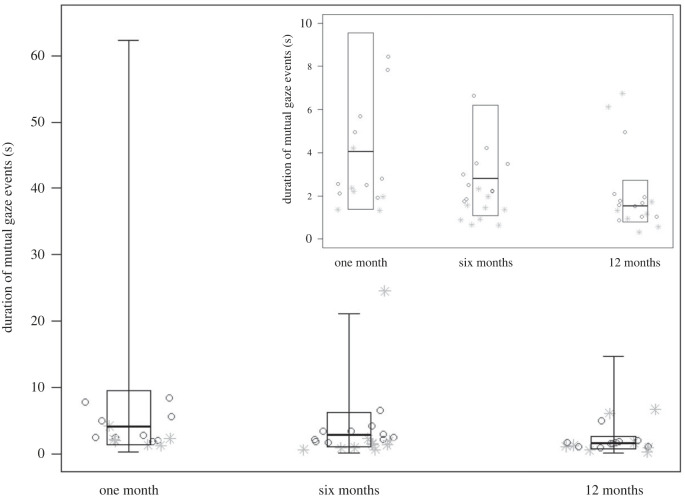
For all study subjects, central lines represent the median duration of mutual gaze events (in seconds) when infants were one, six and 12 months old. The horizontal ends of the box represent the 75% and 25% quartiles. The ends of the whiskers represent the 97.5% and 2.5% quartiles, and are not depicted in the smaller panel (which is otherwise identical to the larger one), to visually facilitate the detection of differences across developmental phases. Dark grey circles and light grey asterisks represent individuals’ median duration of mutual gaze events at different developmental phases, for WEIRD urban humans and for captive zoo-based chimpanzees, respectively. Overall, mutual gazes were longer at one month than at six and 12 months of age (model 2).

In model 2, we tested whether the duration of mutual gazes was affected by context, and by the two-way interaction of study group and age. The full model was significantly better than the null one (GLMM, *χ*^2^_11_= 43.03, *p* < 0.001), with study group (*p =* 0.012), age (*p =* 0.001) and context (*p =* 0.015) having a significant effect as main predictors (table 3). In particular, the duration of mutual gazes was overall longer in WEIRD urban humans than in captive zoo-based chimpanzees. *Post hoc* tests further revealed that the duration decreased in both study groups, being higher at one month than at six and 12 months of age (one versus six months, *p =* 0.024; one versus 12 months, *p <* 0.001; figure 2). Moreover, the duration of mutual gazes was longer during caring and feeding, as compared to locomotion (caring versus locomotion, *p =* 0.010; feeding versus locomotion: *p* = 0.044; see the electronic supplementary material).

**Figure 3.**
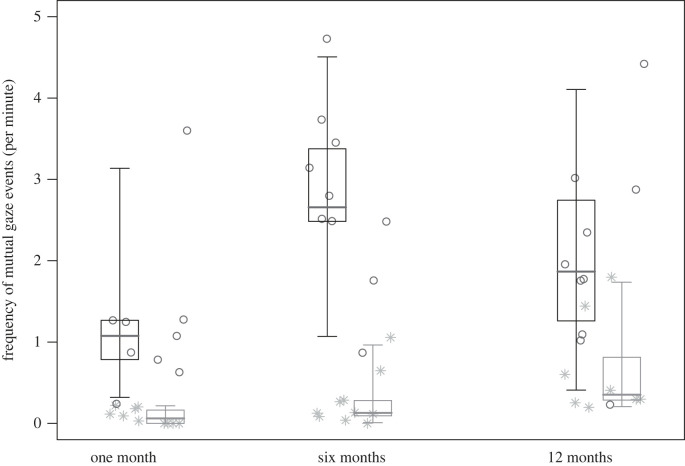
For all study subjects, central lines represent the median frequency of mutual gaze events (per minute) for WEIRD urban humans (in dark grey) and captive zoo-based chimpanzees (in light grey), when infants were one, six and 12 months old. The horizontal ends of the box represent the 75% and 25% quartiles, and the ends of the whiskers represent the 97.5% and 2.5% quartiles. Dark grey circles and light grey asterisks represent individuals’ median frequency of mutual gaze events at different developmental phases, for WEIRD urban humans and for captive zoo-based chimpanzees, respectively. Mutual gazes were more frequent in WEIRD urban humans than in captive zoo-based chimpanzees at all developmental phases; in captive zoo-based chimpanzees, mutual gazes were more frequent at six and 12 months than at one month of age, whereas in WEIRD urban humans they were higher at six than at one month (model 3).

In model 3, we tested whether the frequency of mutual gazes was affected by context, and by the two-way interaction of study group and age. The full and null model significantly differed from each other (GLMM, *χ*^2^_10_= 493.67, *p* < 0.001), with both context (*p <* 0.001) and the two-way interaction between study group and age (*p =* 0.012) having a significant effect (table 3). In particular, *post hoc* tests revealed that the frequency of mutual gazes was significantly higher in WEIRD urban humans than in captive zoo-based chimpanzees at all developmental phases (all *p* ≤ 0.007; figure 3). In captive zoo-based chimpanzees, the frequency of mutual gazes increased through age, being significantly higher at six and 12 months as compared to one month of age (one versus six months, and one versus 12 months, both *p* ≤ 0.049; figure 3). By contrast, the frequency of mutual gazes in WEIRD urban humans peaked at six months (one versus six months: *p =* 0.015; figure 3). Moreover, the frequency of mutual gazes was lower during locomotion than all the other contexts (all *p* < 0.001) and also lower in solitary play than during feeding, resting or social play (*p* < 0.001). Finally, the frequency was higher when resting as compared to caring, feeding and social play (all *p* < 0.001), and when feeding as compared to caring (*p* = 0.032).

## 4. Discussion

Our study showed that face-to-face interactions with mutual gaze events are common in both captive zoo-based chimpanzees and WEIRD urban humans throughout the first year of development, despite variation in quantitative (i.e. duration and frequency) and qualitative aspects (i.e. context in which they occur). Differences between study groups and developmental phases were less clear-cut than we had expected. In particular, although we had predicted that the duration of maternal looks, infants’ looks and mutual gazes would be overall longer, and that the frequency of mutual gazes would be overall higher in WEIRD urban humans than in captive zoo-based chimpanzees (prediction 1), we found mostly no differences between study groups that persisted across all developmental phases. Moreover, although we had predicted that the duration of maternal looks, infants’ looks and mutual gazes, and the frequency of mutual gazes would decrease in both study groups during the first year of development (prediction 2), differences across developmental phases were only partially shared by the two study groups, thus not confirming prediction 2. Instead, we evidenced variation in the developmental patterns of the two groups, with the duration of maternal looks and infants’ looks, and the frequency of mutual gazes, varying through developmental phases in a different way in the two study groups (see below for a more detailed discussion). Finally, as predicted (prediction 3), the duration of looks and mutual gazes, and the frequency of mutual gazes, varied across contexts in both study groups, with events being generally longer and more frequent when mothers engaged in grooming or caring activities, and shorter during locomotion.

Most differences between study groups did not persist across developmental phases: maternal looks were longer in WEIRD urban humans than in captive zoo-based chimpanzees, but only at one month of age, whereas there were no significant differences in the duration of infants’ looks between WEIRD urban humans and captive zoo-based chimpanzees, at any developmental phase (model 1). However, the duration of mutual gaze events was consistently longer in WEIRD urban humans than in captive zoo-based chimpanzees, across all developmental phases (model 2). These results are in line with previous studies on mother–infant interactions, reporting shorter mutual gaze duration in captive zoo-based chimpanzees than in WEIRD humans [33], where mothers often actively promote mutual gaze and face-to-face interactions with their infants [59], in line with cultural values of equality and independence [60,61].

Taken together, these results further suggest that the longer duration of mutual gaze events in WEIRD urban humans does not simply depend on mothers or infants looking longer at each other, but on a better coordination of the gazing behaviour between the two, who are more likely to simultaneously look at each other, as compared to captive zoo-based chimpanzees. In line with this, also the frequency of mutual gaze events was higher in WEIRD urban humans than in captive zoo-based chimpanzees, and this was true at all ages (model 3). In captive zoo-based chimpanzees, the frequency of mutual gazes varied between five (at one month of age) and 40 occurrences every hour (at 12 months of age), and it was therefore in line with previous studies showing a variation between approximately 10 (at one month) up to 30 occurrences per hour (at three months of age), depending on the study group [24]. Furthermore, as for previous studies, we found high variation in terms of duration and frequency of mutual gazes, not only across individual humans, but also across chimpanzees (figures 2 and 3), which suggests large flexibility in individual development and in the way mothers engage with their infants (see [24]). On a descriptive level, moreover, infants initiated most mutual gaze events, looking at mothers when mothers were already looking at them. Although this was true in both study groups, and across all developmental phases, it was especially evident in WEIRD urban humans at 12 months of age, when the proportion of mutual gazes initiated by infants was around four times that initiated by their mothers (table 2). In line with this, we found differences in the behaviour of mothers and infants, but only in WEIRD urban humans at 12 months, when the duration of maternal looks was significantly longer than the duration of infants’ looks (model 1). These results suggest that, in WEIRD societies, face-to-face interactions may be often the result of mothers spending a relatively long time looking at their infants, so that when infants look at their mothers, they are likely to initiate mutual gazes.

Developmental trajectories were only partially similar in our study groups. In WEIRD urban humans, as predicted, the duration of maternal and infants’ looks decreased through development (model 1), as infants became more independent and more often engaged in solitary and social play, directing their looks towards other elements of the environment and only shortly referencing back to their mothers by mutual gaze. However, this was not the case for captive zoo-based chimpanzees, whose duration of maternal and infants’ looks remained similar across developmental phases (model 1). In both study groups, mutual gaze duration decreased through age in a similar way (model 2), but its frequency followed different developmental trajectories, gradually increasing with age in captive zoo-based chimpanzees (in line with [24]), but peaking at six months in WEIRD urban humans (model 3). This study therefore suggests important differences in the two study groups in the temporal emergence of communicative skills during ontogeny, and in particular in the ability (and/or motivation) of mothers and infants to coordinate their looks into mutual gaze events. Moreover, developmental changes in the duration of infants’ looks in WEIRD urban humans might suggest that these patterns are highly flexible, likely to be largely guided by socialization practices and thus to also vary across societies. These differences in developmental patterns highlight the importance of combining a cross-species and developmental approach [1–4,62]. In the future, it will be especially informative to also integrate a cross-cultural approach, by systematically comparing the development of mutual gaze in other human societies. Such a comprehensive approach will allow a better understanding of the properties of face-to-face interactions that humans share across societies, and about the different forms in which they can be instantiated across cultural settings [2,62].

Largely in line with our predictions, we found variation across contexts in the duration of looks and mutual gazes, and in the frequency of mutual gaze in both study groups. The duration of infants’ and maternal looks was generally longest when mothers were grooming or caring for their infants, and shortest when they were in locomotion, as compared to all the other contexts (model 1). Similarly, mutual gaze events were longer (model 2) and partially more frequent (model 3) when mothers engaged in grooming or caring activities with their infants, or when they were feeding, as compared to when they were in locomotion. The frequency of mutual gaze events was also high when mothers and infants were resting, probably reflecting the fact that mothers, in humans, often looked towards their infants when they were not engaged in other activities, even moving the infant's face to facilitate the occurrence of mutual gazes. These results suggest that both captive zoo-based chimpanzees and WEIRD urban humans can flexibly adapt the duration of their gazing patterns to the specific context. On a descriptive level, mutual gazes were also mostly exchanged when mothers and infants were in body contact, suggesting that mutual gaze might often be the ‘by-product’ of the mother's bodily actions on the infant, such as grooming. However, our results are in contrast with two previous studies on humans and captive chimpanzees, which found that mutual gaze decreased with physical contact (e.g. when mothers cradled their infants) in both study groups [24,63]. One possible reason for this difference is the way in which we coded frequencies in our study (i.e. whenever both faces were simultaneously visible and mutual gazes occurred, we coded the context in which it happened and whether individuals were in body contact). In our study, therefore, mutual gazes might have occurred more when individuals were in body contact, simply because it was more likely that both faces were visible. Thus, our findings about contextual variation in the frequency (but not duration) of mutual gazes should be taken with special caution. In the future, it will be especially informative to thoroughly analyse the activities in which mothers and infants engage during mutual gaze events, and how patterns of mutual gaze develop in the different contexts.

Overall, our study contributes to the comparative study of developmental trajectories of mutual gaze and face-to-face interactions in human and chimpanzee groups (see [24]). However, much more work is needed to fully understand differences and similarities in the developmental trajectories of these behaviours. First, human societies strongly differ in the way mothers interact with their infants. In some cultural contexts, for instance, mothers largely rely on a proximal parenting style, in which infants are often in body contact with their carers, while face-to-face interactions seem to play a less relevant role, as compared to WEIRD cultures [15,64]. Therefore, future studies should ideally include humans from different, non-WEIRD societies to better capture this essential intraspecific cultural variation in the way mothers interact with their children. Second, also captive zoo-based chimpanzees might importantly vary in their maternal styles, depending on their life histories and on the social dynamics of the group. Mutual gaze events, for instance, might be more common or longer in captive zoo-based chimpanzees who have had more extensive contact with humans during their development, like nursery-reared chimpanzees [24,65]. Therefore, future studies should include a larger number of mother–infant pairs from different settings, possibly also including wild chimpanzees. Third, future comparisons would largely benefit from including more qualitative measures, including the occurrence of other interactions during mutual gazes (e.g. facial touches) or the mirroring of facial expressions as a proxy of mutual engagement and affect sharing [64]. In this regard, the use of detailed analyses of facial movements (e.g. by using the Facial Action Coding System) and its modified versions for coding non-human species, see www.animalfacs.com, might open exciting avenues for future research.

Our study evidenced several similarities in the behaviour of WEIRD urban humans and captive zoo-based chimpanzees during face-to-face interactions, but also some differences in the frequency and duration of mutual gazes, which appeared to be more relevant for humans, especially in the first months of infants’ development, and probably the result of better coordination between mothers and offspring. Therefore, our results confirm that several aspects of early socio-cognitive development are shared by human and other primate groups (see also [1,33]). Future longitudinal studies combining the developmental, cross-species and cross-cultural perspectives will provide a powerful tool to better understand when these behaviours first appeared in our evolutionary history, the socio-ecological conditions that facilitate their emergence, and the richness resulting from the different ways in which they can be moulded across cultural settings.

## Data Availability

Data and code are available at the Open Science Framework: https://doi.org/10.17605/OSF.IO/9FNKR [66]. The data are provided in the electronic supplementary material [67].
